# Variants in *COMT*, *CYP3A5*, *CYP2B6,* and *ABCG2* Alter Quetiapine Pharmacokinetics

**DOI:** 10.3390/pharmaceutics13101573

**Published:** 2021-09-28

**Authors:** Pablo Zubiaur, Paula Fernández-Campos, Marcos Navares-Gómez, Paula Soria-Chacartegui, Gonzalo Villapalos-García, Manuel Román, Gina Mejía-Abril, Dolores Ochoa, Francisco Abad-Santos

**Affiliations:** 1 Clinical Pharmacology Department, La Princesa University Hospital, Instituto Teófilo Hernando, Instituto de Investigación Sanitaria La Princesa (IP), Universidad Autónoma de Madrid (UAM), 28029 Madrid, Spain; paula.fcampos@alumnos.upm.es (P.F.-C.); marcos.navares@salud.madrid.org (M.N.-G.); paulasch98@gmail.com (P.S.-C.); g.villapalos@salud.madrid.org (G.V.-G.); manuel.roman@salud.madrid.org (M.R.); ginapaola.mejia@scren.es (G.M.-A.); mdolores.ochoa@salud.madrid.org (D.O.); 2 UICEC Hospital Universitario de La Princesa, Plataforma SCReN (Spanish Clinical Research Network), Instituto de Investigación Sanitaria La Princesa (IP), 28006 Madrid, Spain; 3 Centro de Investigación Biomédica en Red de Enfermedades Hepáticas y Digestivas (CIBERehd), Instituto de Salud Carlos III, 28209 Madrid, Spain

**Keywords:** quetiapine, pharmacogenetics, pharmacokinetics, safety, precision medicine

## Abstract

Quetiapine is an atypical antipsychotic widely used for the treatment of schizophrenia and the depressive episodes of bipolar disorder. The aim of this work was to investigate the effect of variants in relevant pharmacogenes in the pharmacokinetics of quetiapine and to exploratorily evaluate adverse drug reaction (ADR) incidence based on genetic polymorphism. Specifically, 49 healthy volunteers enrolled in two bioequivalence clinical trials were included in this study. In addition, 80 variants in 19 relevant pharmacogenes were genotyped, including cytochrome P450 (CYP) genes, catechol-O-methyl transferase (*COMT*), other enzymes (e.g., *UGT1A1* or *UGT1A4*), and transporters (e.g., *SLCO1B1, ABCB1*, or *ABCG2*). The *COMT* rs13306278 T allele was significantly related to quetiapine-increased exposure. We demonstrated the existence of quetiapine derivatives with a catechol-like structure (7,8-dihydroxi-quetiapine and 7,8-dihydroxi-N-desalkyl-quetiapine), which would be COMT metabolites and would explain quetiapine accumulation through CYP2D6 and CYP3A4 negative feedback. Moreover, CYP3A5 and *CYP2B6* phenotypes were related to quetiapine exposure variability, which confirms (for CYP3A5) and suggests (for CYP2B6) that these enzymes play an important role in quetiapine’s metabolism. Finally, the *ABCG2* rs2231142 T allele was related to quetiapine accumulation. Further studies are required to confirm the clinical relevance of our findings.

## 1. Introduction

Quetiapine is an atypical antipsychotic widely used for the treatment of schizophrenia and the depressive episodes of bipolar disorder [1]. Although its precise mechanism of action remains controversial, it is a serotonin 5-HT2 receptor antagonist (HTR2) and a dopamine D1 and D2 receptor antagonist (DRD1 and DRD2), with affinity for other receptors such as histamine H1, muscarinic M1, M3, and M5, and α1-adrenergic and other serotonin receptors. It also inhibits the norepinephrine transporter (NET). Blockade of DRD2 in the mesocortical and mesolimbic pathways is proposed as the interaction responsible for the treatment of schizophrenia, where increased dopamine levels are responsible for negative and positive symptoms, respectively. 5-HT2 antagonism is related to quetiapine’s antidepressant activity [2].

It is orally administrated in tablets or solution and presents a rapid absorption, not affected by food intake, with the time to reach the maximum concentration (C_max_) being 1 to 2 h (t_max_). It shows linear pharmacokinetics. However, due to extensive first-pass metabolism, quetiapine has a poor absolute oral bioavailability of 9% [3]. Metabolism is mainly hepatic, by cytochrome the P450 (CYP) 3A4 isoform [4]. In addition, CYP2D6 [1] and CYP3A5 [5] can contribute to the metabolism of quetiapine. CYP3A4 is responsible for the transformation of quetiapine into N-desalkyl quetiapine, also known as norquetiapine, which is the most abundant active metabolite. Two additional active metabolites are formed: 7-hydroxy quetiapine, obtained after direct hydroxylation of quetiapine by CYP2D6, and 7-hydroxy-N-desalkyl quetiapine, obtained after the hydroxylation of norquetiapine also by CPY2D6 [6]. Furthermore, 83% of the drug in the blood is bound to plasma proteins, and its elimination half-life (t_1/2_) is approximately 7 h [1]. Quetiapine excretion mainly occurs through urine (73%) and feces (21%) [1]. Quetiapine is a substrate of the transmembrane multidrug resistance transporter P-glycoprotein (P-gp), which is codified by the *ABCB1* (ATP Binding Cassette, Family B, member 1) gene. This active transporter (ATP-dependent), which is located in the blood–brain barrier, influences blood–brain barrier permeability and, consequently, the access of the drug to the brain [7]. Moreover, the P-glycoprotein plays an important role in the pharmacokinetic processes of several drugs, as it participates in their absorption, distribution, metabolism (indirectly, by conditioning the access to metabolizing organs such as the liver), and elimination [8]

Despite atypical antipsychotics improving the tolerability of classic or typical antipsychotics, they are not innocuous. Indeed, they can produce considerable side effects that can condition treatment adherence. The most notable adverse drug reactions (ADRs) related to quetiapine intake are somnolence (25–39%), dizziness (15–27%), headache (10–23%), hypotension (6–18%), and metabolic effects such as weight gain (11–30%) [9]. They are typically dose-dependent, except the last one, which belongs to the class of metabolic effects, which are considered dose-independent and require a prolonged exposure to the drug [10].

Pharmacogenetics studies the impact of genetic variants in the response to drugs. To date, no pharmacogenetic guideline has been published recommending a quetiapine dose adjustment based on the patient’s genetic polymorphism. Moreover, despite some pharmacogenetic studies being published [11,12], no consensus has been reached to date on the clinical relevance of such polymorphisms, i.e., the effects on therapy effectiveness and safety. This work aimed to investigate the effect of relevant variants in relevant pharmacogenes (e.g., metabolizing enzymes such as CYPs, ABC, or SLC transporters, or other enzymes such as COMT or UGT), along with demographic characteristics, in the pharmacokinetics of quetiapine; furthermore, we aimed to exploratorily evaluate ADR incidence based on genetic polymorphism. 

## 2. Materials and Methods

### 2.1. Study Design and Population

The data for the candidate gene pharmacogenetic study were obtained from two bioequivalence clinical trials performed at the Clinical Trials Unit of Hospital Universitario de La Princesa (UECHUP): clinical trial 1, EUDRA-CT 2018-003079-37, and clinical trial 2, EUDRA-CT 2020-001091-14. Both were randomized, open-label, one-center, crossover bioequivalence clinical trials of two quetiapine formulations after a single oral dose administration to healthy volunteers. In both clinical trials, the reference formulation (R) was Seroquel^®^ 25 mg film-coated tablets (AstraZeneca Farmacéutica Spain, Madrid, Spain). The test formulation (T) of clinical trial 1 was quetiapine fumarate oral suspension 25 mg/mL, and quetiapine 50 mg film-coated tablets were the test formulation for clinical trial 2. Clinical trial 1 had two sequences (RT and TR) and two periods. In either period, volunteers were randomly assigned to receive a quetiapine formulation, and, in the subsequent period, they received the other one. Clinical trial 2 had a replicated design, and the quetiapine dose was 50 mg (2 tablets of 25 mg for R, 1 tablet of 50 mg for T). In each period, volunteers were randomly assigned to receive either formulation, ensuring that each volunteer received each formulation twice, for a total of four periods. The organization of the periods was designed in four sequences (RTRT, TRTR, RTTR, and TRRT). Only the reference formulation in either clinical trial was considered for this pharmacogenetic study. Mean pharmacokinetic parameters of the reference formulation for each subject were calculated to reduce variability in the replicated study.

Both clinical trials (Project code: EUDRA-CT: 2020-001091-14, date of IEC approval: September 24, 2020, IEC code: 4252; Project code: EUDRA-CT: 2018-003079-37, date of IEC approval: November 22, 2018. IEC code: 3592) were approved by the Independent Ethics Committee on Clinical Research (IECCR) of the Hospital La Princesa and the Spanish Drug’s Agency (AEMPS). They were conducted in accordance with Spanish legislation and they followed the International Conference on Harmonization-Good Clinical Practice (ICH-GCP) guidelines and the Revised Declaration of Helsinki [13,14]. A number of 36 healthy volunteers, who fulfilled the inclusion criteria, provided their informed consent to participate in each bioequivalence clinical trial. Of the total number of subjects (*n* = 72), 49 consented for participation in the pharmacogenetic study, which was likewise independently approved by the IECCR. 

The inclusion criteria included: males or females, aged from 18 to 55, free from organic or psychic conditions, with normal medical, physical, and laboratory records. Exclusion criteria comprised: use of any type of pharmacological treatment two days before hospitalization, use of prescription treatments in the last 15 days (except for women using contraceptives), body mass index outside the 18.5–30 kg/m^2^ range, history of sensitivity to any drug, positive drug screening, alcohol poisoning in the week before hospitalization, smoking, having donated blood in the last month before hospitalization, pregnant or breastfeeding women, participation in another study with the administration of investigational drugs in the previous 3 months, inability to collaborate during the study, lactose intolerance, galactose intolerance, Lapp lactase deficiency or glucose-galactose malabsorption, and history of swallowing difficulty.

### 2.2. Pharmacokinetics and Safety

Several EDTA-K2 blood tubes were extracted for pharmacokinetic profiling, i.e., the quantification of quetiapine plasma concentrations and concentration-time curve tracing. On each period of clinical trial 1, each subject provided 17 blood samples at the following times: baseline (before receiving the drug), 0.17 h, 0.33 h, 0.5 h, 0.75 h, 1 h, 1.5 h, 2 h, 2.5 h, 3 h, 4 h, 5 h, 6 h, 8 h, 10 h, 12 h, and 24 h after the administration of each of the formulations. On each period of trial 2, each volunteer provided 16 blood samples at the following times: baseline, 0.33 h, 0.67 h, 1 h, 1.5 h, 2 h, 2.5 h, 3 h, 4 h, 5 h, 6 h, 8 h, 10 h, 12 h, 24 h, and 48 h after drug intake. After centrifugation, all plasma samples were stored at −20 °C (±5 °C). The determination of the drug plasma levels was performed by an external analytical laboratory with high-performance liquid chromatography coupled to a tandem mass spectrometer (HPLC-MS/MS); this method was validated according to the European Medicines Agency’s standards, with a lower limit of quantification of 0.5 ng/mL.

The following pharmacokinetic parameters were directly obtained from the plasma concentration–time curves: the quetiapine maximum plasma concentration (C_max_) and the time it lasted to reach it (t_max_). The Area Under the Curve at time t (AUC_0–t_) was calculated with the program WinNonlin Professional Edition version 8.3 (Scientific Consulting, Inc., Cary, NC, USA) according to the linear trapezoidal rule, which is based on a noncompartmental model. The extrapolation to infinity (AUC_0–∞_) was determined by adding two partial AUCs: AUC_0–t_ and AUC_t–∞_, which was calculated as the C_t_/K_e_ ratio, with Ct being the last detectable concentration and K_e_ the constant of elimination (i.e., the slope of the line obtained by linear regression from the points corresponding to the drug’s elimination phase). In addition, the elimination half-life (t_1/2_) was estimated as –ln 2/k_e_. Drug clearance adjusted for bioavailability (Cl/F) was calculated as the dose divided by AUC_0–∞_ and corrected for weight (W) (i.e., D/AUC*W) (Cl/F_w_), and the volume of distribution adjusted for bioavailability (Vd/F) was calculated as Cl/F divided by K_e_, and corrected for W (Vd/F_w_).

The evaluation of safety and the identification of adverse events (AEs) were performed by means of open questions to the volunteers, physical examination, vital signs monitoring, including a 12-lead electrocardiogram (1.5 and 5 h after drug intake), and by serum, urine, and biochemistry analyses. The determination of causality was carried out with the Spanish Pharmacovigilance System algorithm [15]. Only those AEs with a possible, probable, or definitive relationship with quetiapine intake were classified as adverse drug reactions (ADRs) and considered for the present study.

### 2.3. Genotyping, Haplotyping, and Phenotyping

DNA was extracted from peripheral blood in a Maxwell^®^ RSC Instrument (Promega Biotech Ibérica S.L., Alcobendas, Madrid, Spain). Genotyping was performed by real-time quantitative polymerase chain reaction (qPCR) with TaqMan^®^ hydrolysis probes. To achieve this, a QuantStudio 12K Flex qPCR instrument (Applied Biosystems, ThermoFisher, Waltham, MA, USA) was used. An OpenArray thermal block and a customized array were used to genotype the variants shown in Table 1. Furthermore, a *CYP2D6* gene copy number assay (CNV) was performed in the same instrument with a 96-well thermal block.

Phenotypes were inferred based on the obtained genotypes. *CYP2B6* (*4, *5, *6, *7, *9, *18, and *22), *CYP2C19* (*2, *3, *4, *5, *6, *7, *8, *9, *17, and *35), *CYP2C9* (*2, *3, *5, *8, and *11), *CYP2D6* (*3, *4, *5, *6, *7, *8, *9, *10, *12, *14, *15, *17, *19, *29, *41, *56B, *59, and CNVs), *CYP3A5* (*3, *6, and *7), and *UGT1A1* (*6 and *80) alleles were used to assign the enzyme phenotype based on CPIC guidelines [16,17,18,19,20,21]. *SLCO1B1* alleles (*2, *3, *5, *6, *9, *10, and *17) were used to infer the transporter’s phenotype following CPIC’s guidance [22]. Variants in the remaining genes were individually analyzed (*CYP1A2*, *CYP2A6*, *CYP2C8*, *CYP3A4*, *ABCB1*, *ABCC2*, *ABCG2, COMT*, *SLC22A1*, *SLC28A3*, *UGT1A4*, and *UGT2B15*) as no information on phenotype inference or allele definition is properly defined yet.

### 2.4. Mass Spectrometry

To demonstrate the existence of quetiapine catechol metabolites, mass spectrometry was used. All available samples of volunteers heterozygous for *COMT* rs13306278 (*n* = 3) were selected and matched with three *COMT* rs13306278 wildtype volunteers with different CYP2D6 phenotypes. The MS signal at t = 2 h and t = 10 h post-dose was determined to address abundance variation. An Agilent instrument consisting of a 1200 Series HPLC module and a triple quadrupole 6410B mass spectrometer (Agilent Technologies, Santa Clara, CA, USA), with positive mode ESI, was used for the screening of quetiapine catechol metabolites. The Agilent MassHunter Workstation Data Acquisition software was used. Plasma was extracted with protein precipitation with 0.1% formic acid acetonitrile. After centrifugation, 25 microliters were directly injected with the HPLC system without an analytical column, with a 50:50 water-ACN isocratic mobile phase for 1 min. To eliminate phospholipid signals, a solid-phase extraction method was used following our previously published methodology [23]. Samples were run initially in MS2-scan mode to identify analyte peaks and, subsequently, in MS2-sim mode, to quantify analyte abundance.

### 2.5. Statistical Analysis

Statistical analysis was carried out with SPSS software (version 21.0, SPSS Inc., Chicago, IL, USA). Only the pharmacokinetic data of the reference product, Seroquel^®^, were used for the statistical analysis. As the dosage of quetiapine was different between the studies (25 mg in clinical trial 1 and 50 mg in clinical trial 2), AUC_0–∞_ and C_max_ were divided by the dose/weight (DW) ratio. All pharmacokinetic parameters were logarithmically transformed in order to normalize their distributions.

Statistical significance was considered *p* < 0.05. An initial descriptive analysis of demographic characteristics was performed. Regarding quetiapine pharmacokinetics, an initial univariate analysis was performed, where all pharmacokinetic parameters were evaluated based on demographic characteristics (e.g., sex or race) or genetic variables (i.e., phenotypes or genotypes). For the comparison of means according to variables with two categories, a *t*-test was used (e.g., age according to sex), while for variables with 3 or more categories, an ANOVA test was used followed by a Bonferroni post hoc (e.g., C_max_/DW according to CYP2D6 phenotype). Further, a multivariate analysis of each pharmacokinetic parameter was performed, with the independent variables being those with *p* < 0.10 in the univariate analysis, as well as sex and race, which were introduced as categorical covariates for all analyses. For this purpose, multiple linear regression was used. A similar methodology was used for the analysis of safety. Initially, a univariate analysis was carried out comparing the incidence of ADRs according to demographic characteristics and genetic variables. For this purpose, Pearson’s chi-squared test was used, unless more than 20% of cells had expected frequencies lower than 5; in these cases, Fisher’s exact test was used. Moreover, following the same methodology as for pharmacokinetics, logistic regression was used to perform the multivariate analysis of ADR. In both multivariate analyses, a Bonferroni correction for multiple comparisons was performed, correcting the threshold for significance (*p* = 0.05) by the number of comparisons.

## 3. Results

A total of 37 men and 32 women completed the bioequivalence clinical trials. The majority of them (54) were Latino-Americans, while 18 reported to be Caucasian. Women had a lower weight and height than men did (*p* < 0.05), while no significant difference was observed for body mass index (BMI) or age according to sex (Table 2). No significant differences in the demographic characteristics were observed based on race either (Table 2).

Here, 69 volunteers (37 men and 32 women) received the reference formulation. No significant differences were observed in uncorrected AUC_0–∞_ and C_max_ between males and females. These parameters were 247.6 ng h/mL and 81.6 ng/mL in clinical trial 1 and 539.2 ng h/mL and 143.0 ng/mL in trial 2 (*p* < 0.001 and *p* < 0.001, respectively) (*n* = 69).

Volunteers from clinical trial 2 exhibited a higher t_1/2_ than those from clinical trial 1 (5.24 h vs. 4.57 h, respectively, *p* = 0.041). Likewise, t_1/2_ was higher in Latino-American volunteers compared to Caucasians (*p* = 0.038, unstandardized β coefficient = 0.172, and R^2^ = 0.375) (*n* = 49) (Table 3).

Table 4 shows the significant associations between pharmacokinetic parameters and the genotypes or phenotypes (*n* = 49). Subjects with the *COMT* rs13306278 C/T genotype had higher AUC_0–∞/_DW (*p* = 0.008) and C_max_/DW (*p* = 0.035) and lower values of Vd/F_w_ (*p* = 0.017) and Cl/F_w_ (*p* = 0.007) in comparison with C/C subjects. CYP2B6 PMs showed higher t_1/2_ compared to carriers of other phenotypes (RM + NM + IM) (*p* = 0.015). Individuals with the CYP3A5 PM phenotype were significantly related to higher t_1/2_ in comparison to carriers of the other phenotypes (NM + IM) (*p* = 0.018) and a tendency was observed toward higher AUC_0–∞/_DW and lower Cl/F_w_ (*p* = 0.065 and 0.066, respectively). In addition, individuals with the UGT1A1 PM phenotype presented lower t_max_ than those with the NM phenotype (*p* = 0.049). The remaining genotypes or phenotypes showed no association with the variability in quetiapine pharmacokinetic parameters in univariate analysis (data not shown). Supplementary Table S1 shows pharmacokinetic parameters based on CYP2D6 phenotype. Moreover, all subjects were *CYP3A4* * 1/* 1 except for one * 1/* 22 and one * 1/* 3 carrier. The latter exhibited an AUC/DW of 1455.04 kg ng h/mL mg and a t_1/2_ of 8.42 h, considerably higher than the mean of other subjects.

In the multivariate analysis, sex, race, *ABCG2* rs2231142 (*p* < 0.10), *COMT* rs13306278, CYP2B6 phenotype, CYP3A5 phenotype, and UGT1A1 phenotype were used as independent variables. *COMT* rs13306278 C/T was again related to higher AUC_0–∞_/DW (*p* = 0.008, unstandardized β coefficient = 0.377, and R^2^ = 0.142), higher C_max_/DW (*p* = 0.035, unstandardized β coefficient = 0.302, and R^2^ = 0.091) and lower Cl/F_w_ (*p* = 0.007, unstandardized β coefficient = −0.378, and R^2^ = 0.143) compared to *COMT* rs13306278 C/C. Moreover, CYP2B6 PMs, CYP3A5 PMs, *ABCG2* rs2231142 T/T carriers, and Latino-Americans were related to t_1/2_ variability (*p* = 0.005, 0.004, 0.027, and 0.038, respectively; unstandardized β coefficients = 0.35, 0.362, −0.275, and 0.258, respectively; R^2^ = 0.375) compared to CYP2B6 RMs + NMs + IMs, CYP3A5 NMs + IMs, *ABCG2* rs2231142 G/G + G/T individuals, and Caucasians, respectively. Additionally, *COMT* rs13306278 C/T subjects, *ABCG2* rs2231142 T/T individuals, and UGT1A1 PMs were associated with Vd/F_w_ variability (*p*-values = 0.005, 0.003, and 0.014, respectively; unstandardized β coefficients = −0.365, −0.392, and 0.32, respectively; R^2^ = 0.323) compared to *COMT* rs13306278 C/C, *ABCG2* rs2231142 G/G+G/T individuals, and UGT1A1 NMs + IMs (Table 2 and Table 3). Furthermore, UGT1A1 PMs were related to lower t_max_ (*p* = 0.018, unstandardized β coefficient = −0.336, and R^2^ = 0.113) compared to UGT1A1 NMs + IMs. Finally, after applying Bonferroni correction in the multivariate analysis, the level of significance was set at *p* < 0.007. *COMT* rs13306278 still remained significant for Vd/F_w_ and Cl/F_w_, CYP2B6, and CYP3A5 phenotypes for t_1/2_ and *ABCG2* rs2231142 for Vd/F_w_. None of the genotypes or phenotypes shown in Table 4 showed unequal distributions according to race (*p* > 0.05 in all cases). Pharmacokinetic parameters based on CYP2D6 phenotype are shown in supplementary Table S1. These parameters, based on the remaining genotypes or phenotypes without statistically significant associations, are shown in supplementary Table S2.

### 3.1. Safety

A total of 29 ADRs were reported (10 in clinical trial 1 and 18 in clinical trial 2) by 22 volunteers, 11 of which were men, 11 women; 3 of them were Caucasians and 19 Latino-Americans. Nineteen volunteers suffered only one ADR, and the remaining three subjects suffered three ADRs each. Dizziness was reported in 7 occasions, nausea or vomiting 4 times, headache 4 times, decreased blood pressure was evidenced 3 times, and arrythmia 11 times (which included five cases of pre-syncope, three cases of tachycardia, one of palpitations, one of atrioventricular junctional rhythm, and one of first-grade atrioventricular block). No significant differences in ADR incidence were observed based on sex or race.

Volunteers from clinical trial 2 were related to a higher arrhythmia incidence (8 out of 36, 22.2%) compared to those from clinical trial 1 (0 out of 36, 0%) (*p* = 0.005). *COMT* rs4680 A/A subjects were related to decreased blood pressure (2 out of 11, 18.2%) compared to G/G (0 out of 26, 0%) and G/A (0 out of 12, 0%) genotypes (*p* = 0.047); finally, the SLCO1B1 phenotype determined the incidence of nausea and vomiting: normal function (NF): 0 out of 28 (0%), decreased function (DF): 1 out of 16 (6.3%), and PF: 1 out of 3 (33.3%) (*p* = 0.047). None of these associations were observed after multivariate analysis, which showed the following other ones: volunteers with the *ABCG2* rs2231142 T/T genotype were associated with a higher risk for presenting nausea (log OR = 3.78, *p* = 0.03) and decreased blood pressure (log OR = 3.78, *p* = 0.03); a lower quetiapine t_1/2_ was related to a higher risk for dizziness (log OR = −6.46 ln*h, *p* = 0.022). After applying Bonferroni correction for multiple comparisons, all these associations disappeared. 

### 3.2. Mass Spectrometry Analysis

Six volunteers were selected based on CYP2D6 phenotype and *COMT* rs13306278 genotype in order to explore the presence of quetiapine-derived catechol metabolites at t = 2 h and t = 10 h (refer to the discussion section for a detailed explanation). Table 5 shows the drop-in analyte abundance between both time points. The proposed catechol metabolites 7,8-dihydroxi-quetiapine and 7,8-dihydroxi-N-desalqyl-quetiapine were identified (m/z: 416.4 and 328.3, respectively) and their abundance was comparable to that of quetiapine and previously known metabolites. No significant differences in analyte drop were observed according to CYP2D6 phenotype and *COMT* rs13306278 genotype.

## 4. Discussion

Genetics might be one of the factors that condition the response to quetiapine, as shown in preceding studies [11,12,24,25]. Hence, observational pharmacogenetic studies such as the present work contribute to generate more scientific evidence. This work intended to provide a deeper insight into the interaction between genetic polymorphisms and the pharmacokinetics and adverse reactions of quetiapine.

On the basis of our results, and congruent with the literature [1], the linear pharmacokinetics of quetiapine was confirmed as the AUC_0–∞_ and C_max_ values without DW correction were approximately double in clinical trial 2, which also presented double the quetiapine dose (50 mg) in comparison to the first clinical trial (25 mg). In addition, AUC and C_max_ values from clinical trial 1 coincide with those described in the literature after a 25 mg single-dose administration to healthy volunteers: 248–366 ng h/mL and 53–86.8 ng/mL [1]. The differences in t_1/2_ according to the clinical trial (univariate analysis) and race (multivariate analysis) were lost after Bonferroni correction for multiple comparisons; therefore, they may be spurious.

Consistent with DPWG guidelines, CYP2D6 had no impact on quetiapine’s pharmacokinetic variability (Supplementary Table S1) [26]

The most relevant and surprising finding was the association between *COMT* rs13306278 C/T genotype and the increased quetiapine exposure and reduced clearance and volume of distribution. Not only did we control bias by performing univariate and multivariate analyses and even a Bonferroni correction for multiple comparisons, but we also investigated possible confounding factors specific to this association. Based on such novel results, a thorough discussion of them was warranted. The *COMT* gene codifies for catechol O-methyl transferase, an enzyme that catalyzes the degradation of catecholamines, including dopamine [27]. Hence, COMT regulates dopamine bioavailability, which is a neurotransmitter with relevance in psychotic diseases. *COMT* polymorphisms were significantly and frequently associated with schizophrenia development [27] and the effectiveness and safety of antipsychotic treatment [12], including quetiapine and other drugs, such as risperidone and olanzapine [28,29]. Moreover, it participates as a phase II enzyme in the metabolism of some drugs such as the antidepressant paroxetine [30]. 

**Figure 1:**
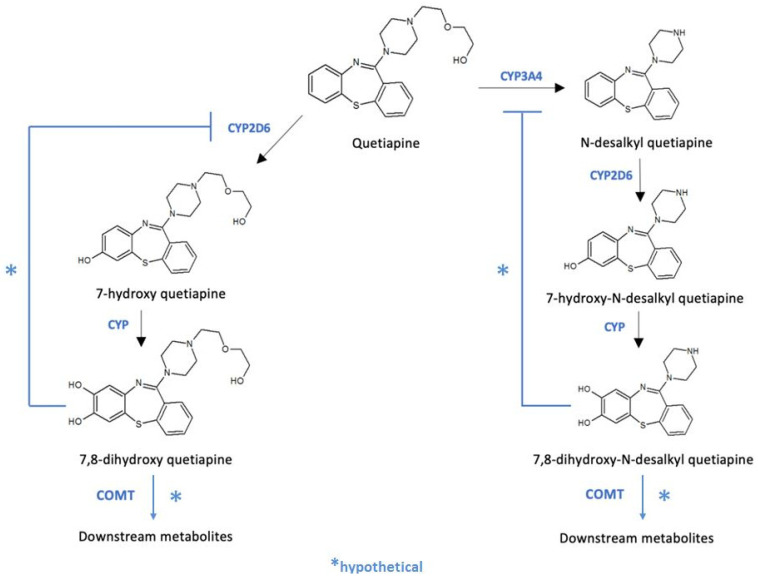
Hypothetical formation of the quetiapine catechol-containing metabolites 7,8-dihydroxy quetiapine and 7,8-dihydroxy-N-desalkyl quetiapine, downstream COMT metabolism, and inhibition by negative feedback of CYP2D6 and CYP3A4 enzymes. CYP: cytochrome P450. CYP2D6: cytochrome P450, 2D6 isoform. CYP3A4: cytochrome P450, 3A4 isoform. Figure created with ChemSketch software (ACD/Labs, Toronto, ON, Canada).

**Figure 2:**
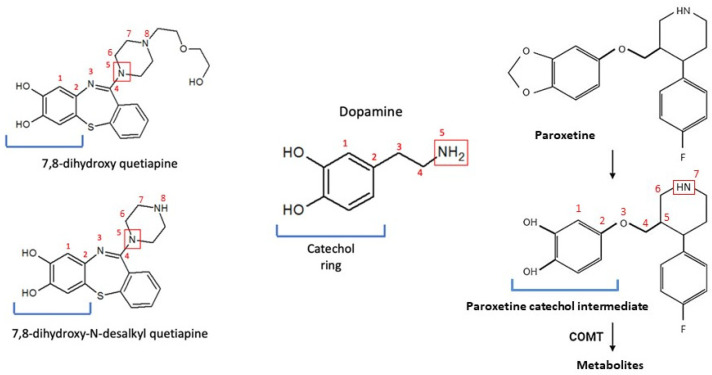
Comparison of catechol structures between 7,8-dihydroxy quetiapine, 7,8-dihydroxy-N-desalkyl quetiapine, dopamine, and paroxetine catechol intermediate. Figure created with ChemSketch software(ACD/Labs, Toronto, ON, Canada).

In previous studies with psychiatric patients, *COMT* rs5993883, rs6269, and rs4818 mutant alleles were associated with better quetiapine response [12]. The hypothesis behind these associations was that the alteration of endogenous dopamine metabolism by the COMT enzyme predisposes the efficacy of antipsychotics [12]. In our work, quetiapine accumulation was related to *COMT* rs13306278 genotype. This is an intronic variant that could have an impact on *COMT* expression and splicing. Thus, an alternative hypothesis arises: COMT might contribute to quetiapine phase II metabolism. Notably, quetiapine oxidation by CYP2D6 in phase I metabolism produces 7-hydroxy quetiapine and 7-hydroxy-N-desalkyl quetiapine [31], both active metabolites, which could be further hydroxylated at position 8, generating 7,8-dihydroxy quetiapine and 7,8-dihydroxy-N-desalkyl quetiapine. After this hydroxylation, a catechol ring would be formed, being possible substrates of COMT (Figure 1). Indeed, these hypothetical metabolites resemble the catecholamine structure due to the presence of a nitrogen atom with five positions of distance to one of the hydroxyl groups of the ring (Figure 2). Assuming that *COMT* rs13306278 causes a decrease in COMT activity, the accumulation of the metabolites would lead to the inhibition, through negative feedback, of CYP2D6 and CYP3A4, causing quetiapine accumulation (Figure 1). Congruently, the two volunteers who suffered from decreased blood pressure presented the *COMT* rs4680 A/A (mutant) genotype. It is known that this variant could also contribute to a reduction in COMT function [32,33], producing the same effect described previously: quetiapine would accumulate and the risk for ADRs would be greater. 

Therefore, we set out to demonstrate the existence of these catechol metabolites using mass spectrometry. For this purpose, we selected all available samples of volunteers heterozygous for *COMT* rs13306278 (*n* = 3) and matched them with three wildtype *COMT* rs13306278 volunteers; we aimed, for the latter, to select three volunteers with different CYP2D6 phenotypes, in the case this enzyme had an impact of the catechol metabolite’s clearance. We proposed the measurement of their MS signal at t = 2 h and t = 10 h post-dose to address abundance variation. We reinforced our theory of the existence of catechol metabolites as we observed their hypothetical m/z MS signals. 7,8-dihydroxy quetiapine corresponds to m/z 416.4, with an estimated molecular weight of 415.5 g/mol; 7,8-dihydroxi-N-desalkyl-quetiapine corresponds to m/z: 328.3, with an estimated molecular weight of 327.4 g/mol. In contrast, no significant differences were observed in the metabolite abundance drop between time points according to *COMT* rs13306278 genotype (and CYP2D6 genotype). Therefore, we can suggest that the CYP downstream metabolism of quetiapine metabolites leads to molecules with a catechol structure that are further metabolized by COMT. rs13306278, located in the latter gene, may reduce COMT activity and catechol metabolites would be accumulated, inhibiting CYP enzymes by negative feedback, which would explain quetiapine accumulation. While we were able to demonstrate the existence of catechol metabolites, we could not demonstrate their accumulation based on COMT genotype. Further studies should analyze the complete pharmacokinetic profile of catechol-metabolites and compare their concentrations or AUC, rather than their MS abundances, based on COMT phenotype.

In spite of our theory, the dopaminergic exacerbation due to COMT impairment may occur too. Hence, *COMT* polymorphism could predispose patients to a worse schizophrenia baseline situation but to better prognosis if treated with quetiapine [32]. In order to strengthen our hypothesis, in vitro studies are warranted to demonstrate the existence of catechol metabolites and the proposed interaction. Further, confirmatory studies of metabolite structure (e.g., NMR analysis) would be necessary.

Concerning CYP3A5, PM subjects presented higher t_1/2_ and a tendency (*p* < 0.1) toward elevated AUC/DW compared to NM + IM phenotypes. This is congruent with previous works where CYP3A5*3 was related to an elevated quetiapine AUC and C_max_, in PMs compared to IMs [34]. Further studies are warranted in order to determine if an adjustment of quetiapine dosage based on CYP3A5 phenotype would be required. However, it seems clear that CYP3A5 polymorphism has a relevant impact on quetiapine pharmacotherapy. Consistently, the only carrier of the CYP3A4 * 1/* 3 genotype showed more than twice the mean AUC/DW. Statistical inference was, however, not possible, due to the reduced number of subjects with CYP3A4 variants. Furthermore, to the best of our knowledge, no other study has suggested to date that CYP2B6 functional impairment relates to quetiapine accumulation; our results contrast with previous works, where no effect was observed [35]; however, the latter study may not be a good comparator, as subjects in it received methadone, a well-known CYP2B6 substrate. Our study is the first to suggest that quetiapine could be a CYP2B6 substrate. Further studies should confirm this association. Nonetheless, the fact that t_1/2_ did not vary with total coherence based on CYP2B6 phenotype (decreasing order, PM > RM > NM > IM) suggests this finding could be spurious and should be interpreted cautiously.

The effect of *ABCG2* rs2231142 in some drugs’ exposure is well described. For instance, patients carrying the G/G genotype have significantly lower rosuvastatin concentrations compared to T allele carriers [36,37]. Here, consistently, T/T subjects exhibited approximately twice as much AUC/DW than G/G carriers (not significant), significantly lower t_1/2_ (multivariate analysis) and, even after Bonferroni correction, Vd/F_w_ was significantly lower. It should be noted that, in noncompartmental analysis, Vd/F_w_ derives from Cl/F and Cl/F derives from AUC; therefore, a lower Vd/F_w_ actually signifies a higher exposure or AUC. To the best of our knowledge, this is the first work to relate the *ABCG2* rs2231142 T allele to quetiapine accumulation. Again, additional studies should confirm this association.

Concerning CYP2D6, no associations between its phenotype and quetiapine pharmacokinetics were established. This is consistent with Dutch Pharmacogenetics Working Group’s (DPWG) recommendations on quetiapine, where no CYP2D6 phenotype is considered relevant in regard to quetiapine disposition [26]. Moreover, the impact of CYP3A4 could not be addressed, as no sufficient variability was observed in the variants included. Furthermore, none of the solute carriers (*SLC*) or *ABCB1* transporter genotypes or phenotypes showed significant relationships with pharmacokinetic variability, in accordance to previous studies [38]. Moreover, the remaining enzymes, i.e., CYP2C19, CYP2C9, CYP2C8, also had no impact in quetiapine pharmacokinetics.

Concerning drug safety, this was an exploratory study, where sample size or statistical power were not calculated beforehand; therefore, our conclusions should be considered cautiously. Besides the findings with *COMT* rs4680, the most notable result was that all arrhythmia events occurred in clinical trial 2, where the single dose of quetiapine was 50 mg, doubling the dose of clinical trial 1 (25 mg). Antipsychotic agents are associated with prolongation of the corrected QT interval (QTc), which might result in arrhythmia or syncope in cases of patient overdose [39,40]. Furthermore, it is known that among the most common symptoms when it comes to higher quetiapine doses or poisoning are the cardiovascular ones, namely tachycardia and hypotension [41]. This is in accordance with the staggered way in which quetiapine doses are prescribed. For instance, an schizophrenia adult patient should start with an initial dose of 25 mg twice daily on day 1 with increments of 25–50 mg divided two or three times on days 2 and 3, finally achieving a dose ranging from 150 to 400 mg by day 4 [42]. 

As for the clinical implications of our results, we consider that there is insufficient evidence to date to propose dosage modifications based on the patient’s genotype. We consider *COMT, CYP2B6, CYP3A5*, and *ABCG2* good candidates; however, further studies are warranted. Concerning *CYP2D6*, its polymorphism seems to have no clinically relevant impact.

### Study Limitations

The main limitations of this study are the sample size and the fact that it was performed in healthy volunteers, not allowing the measurement of drug effectiveness. It would be appropriate to increase the sample size in further confirmatory studies in order to gain statistical power and for finding more genetic variability, for example, subjects with the COMT rs13306278 T/T genotype and CYP3A4 variability. Furthermore, the fact that only a single dose of quetiapine was administered impedes the conclusion on the long-term safety of the drug, which includes metabolic effects (e.g., weight gain), which are of considerable relevance in quetiapine treatment. Other limitations are the pharmacokinetics noncompartmental analysis and the array design, which involves the selection of a specific number of polymorphisms for the genotyping. Nevertheless, the study design and the obtained results were robust, as we confirmed the existence of a hypothesized metabolite with mass spectrometry. Lastly, this was an exploratory study, where sample size or statistical power were not calculated beforehand; therefore, our conclusions should be considered cautiously.

## 5. Conclusions

This study describes a novel route of metabolization of quetiapine, not proposed to date. Through the action of CYP enzymes on known metabolites of quetiapine, derivatives with a catechol-like structure would be formed, which would be COMT substrates: 7,8-dihydroxi-quetiapine and 7,8-dihydroxi-N-desalkyl-quetiapine. The *COMT* rs13306278 T allele would cause the functional impairment of the enzyme, and catechol-like metabolites would be accumulated (particularly 7,8-dihydroxi-N-desalqyl-quetiapine), which would inhibit CYP2D6 and CYP3A4 through negative feedback and cause the accumulation of quetiapine. Although the existence of the catechol metabolites was demonstrated, further in vivo and in vitro studies are warranted to demonstrate such negative feedback. Moreover, CYP3A5 and CYP2B6 phenotypes were related to quetiapine exposure variability, which suggest they may play an important role in its metabolism. Finally, this work was the first to suggest that the *ABCG2* rs2231142 T allele is related to quetiapine accumulation. Future studies should be performed to confirm the clinical relevance of our findings.

## Figures and Tables

**Table 1:** Genes, alleles *, and variants analyzed.

Gene	Allele	Variant	Gene	Allele	Variant	Gene	Allele	Variant
*ABCB1*	C3435T	rs1045642	CYP2C8	* 2	rs11572103	*CYP3A4*	* 3	rs4986910
	G2677 T/A	rs2032582		* 3	rs10509681		* 2	rs55785340
	G2677 T/A	rs2032582		* 3	rs11572080		* 6	rs4646438
	C1236T	rs1128503		* 4	rs1058930		* 18	rs28371759
*ABCG2*		rs2231142	*CYP2C9*	* 2	rs1799853		* 22	rs35599367
*ABCC2*		rs2273697		* 3	rs1057910	*CYP3A5*	* 3	rs776746
*COMT*		rs4680		* 5	rs28371686		* 6	rs10264272
		rs13306278		* 8	rs9332094		* 7	rs41303343
*CYP1A2*	* 1C	rs2069514		* 8	rs7900194	*SCL28A3*		rs7853758
	* 1F	rs762551		* 11	rs28371685	*SLC22A1*	* 2	rs72552763
	* 1B	rs2470890	*CYP2D6*	* 3	rs35742686		* 3	rs12208357
*CYP2A6*	* 9	rs28399433		* 4	rs3892097		* 5	rs34059508
*CYP2B6*	* 9	rs3745274		* 6	rs5030655	*SLCO1B1*	* 5	rs4149056
	* 5	rs3211371		* 7	rs5030867		* 1b	rs2306283
		rs4803419		* 8	rs5030865			rs4149015
	* 4	rs2279343		* 9	rs5030656		* 2	rs56101265
	* 22	rs34223104		* 10	rs1065852		* 3	rs56061388
	* 18	rs28399499		* 10	rs1135840		* 6	rs55901008
*CYP2C19*	* 2	rs4244285		* 12	rs5030862		* 9	rs59502379
	* 3	rs4986893		* 14	rs5030865		* 10	rs56199088
	* 4	rs28399504		* 15	rs774671100			rs11045879
	* 6	rs72552267		* 17	rs28371706	*UGT1A1*	* 6	rs4148323
	* 5	rs56337013		* 19	rs72549353		* 80	rs887829
	* 7	rs72558186		* 29	rs59421388	*UGT1A4*		rs2011425
	* 8	rs41291556		* 41	rs28371725	*UGT2B15*		rs1902023
	* 9	rs17884712		* 56B	rs72549347			
	* 17	rs12248560		* 59	rs79292917			
	* 35	rs12769205						

* Alleles are named after tag variants; however, additional alleles were identified with the combination of variants.

**Table 2:** Demographic characteristics of the subjects according to sex and race.

Variable	*N*	Age	Weight (kg)	Height (cm)	BMI
Total	49	31.7 (9.1)	69.2 (13.1)	167.9 (11.2)	24.4 (2.7)
Sex					
Male	28	31.2 (9.1)	75.2 (12.0)	175.0 (9.0)	24.5 (2.8)
Female	21	32.3 (9.8)	61.1 (9.8) *	158.5 (5.4) *	24.2 (2.9)
Race					
Caucasian	9	32.6 (12.6)	70.1 (16.0)	172.0 (14.6)	23.4 (2.9)
Latino-American	40	31.5 (8.6)	69.0 (12.3)	167.0 (10.3)	24.6 (2.7)

Data are shown as mean (standard deviation). * *p* < 0.05 after a *t*-test.

**Table 3:** Pharmacokinetic parameters according to sex, race, and clinical trial design.

Variable	*N*	AUC_0–¥_/DW	C_max_/DW	t_max_ (h)	t_1/2_ (h)	V_d_/F_w_ (L/kg)	Cl/F_w_ (L/h·kg)
		(kg·ng·h/mL mg)	(kg·ng/mL mg)				
Total	49	746.6 (378.7)	201.61 (90.57)	1.34 (1.00)	4.98 (1.18)	12.04 (6.49)	1.78 (1.04)
Sex:							
Male	28	733.09 (379.70)	210.92 (96.83)	1.34 (1.00)	5.01 (0.93)	12.16 (6.07)	1.75 (0.90)
Female	21	764.52 (385.94)	189.20 (82.15)	1.33 (0.95)	4.94 (1.41)	11.89 (7.16)	1.83 (1.23)
Race:							
Caucasian	9	677.87 (409.88)	212.51 (124.11)	1.63 (1.22)	4.57 (1.30)	14.39 (11.05)	2.31 (1.71)
Latino-American	40	762.02 (375.11)	199.16 (83.08)	1.27 (0.91)	5.07 (1.11)	11.52 (5.02)	1.66 (0.81)
Clinical trial:							
1	19	699.87 (306.78)	203.71 (92.80)	1.61 (1.29)	4.57 (1.19)	10.81 (4.69)	1.79 (1.03)
2	30	776.13 (420.26)	200.28 (90.70)	1.17 (0.67)	5.24 (1.06) *	12.83 (7.37)	1.77 (1.07)

Data are shown as mean (standard deviation). * *p* < 0.05 in univariate analysis (*t*-test, *N* = 69). Underlined: *p* < 0.05 in multivariate analysis (with Sex, Race, ABCG2 rs2231142 MUT vs. WT+HTZ, COMT rs13306278 HTZ vs. WT, CYP2B6 PM vs. RM + NM + IM, CYP3A5 PM vs. NM + IM, and UGT1A1 PM vs. NM + IM, *n* = 49). ^$^ *p* < 0.007 after Bonferroni correction.

**Table 4:** Pharmacokinetic parameters according to genotypes and phenotypes showing statistically significant differences.

Genotype or Phenotype	*n*	AUC_0–∞_/DW	C_max_/DW	t_max_ (h)	t_1/2_ (h)	V_d_/F_w_ (L/kg)	Cl/F_w_ (L/h·kg)
		(kg·ng·h/mL·mg)	(kg·ng/mL·mg)				
Total	49	746.56 (378.69)	201.61 (90.57)	1.34 (0.97)	4.98 (1.14)	12.05 (6.49)	1.78 (1.04)
ABCG2 rs2231142:							
G/G	35	686.66 (322.63)	191.4 (86.82)	1.34 (1.01)	5.04 (1.10)	13.23 (7.11)	1.9 (1.09)
G/T	12	855.47 (392.98)	215.57 (95.05)	1.41 (0.94)	5.01 (1.08)	9.65 (2.96)	1.48 (0.85)
T/T	2	1141.53 (1032.36)	296.58 (118.52)	0.79 (0.41)	3.67 (2.25)	5.72 (2.22) ^$^	1.49 (1.33)
COMT rs13306278:							
C/C	42	683.92 (345.07)	190.26 (91.57)	1.28 (0.95)	4.89 (1.09)	12.74 (6.66)	1.91 (1.06)
C/T	7	1122.43 (375.92) *	269.71 (44.91)*	1.67 (1.13)	5.52 (1.41)	7.87 (3.11) *^, $^	1.01 (0.4) *^, $^
CYP2B6 phenotype:							
RM	8	698.71 (354.07)	199.1 (99.02)	1.73 (1.52)	5.44 (1.08)	13.45 (5.81)	1.81 (0.93)
NM	13	701.77 (315.35)	203.28 (87.63)	1.26 (1.01)	4.72 (0.53)	12.6 (8.09)	1.83 (1.12)
IM	23	751.89 (399)	204.64 (96.92)	1.18 (0.68)	4.66 (1.19)	10.7 (4.57)	1.76 (1.03)
PM	5	915.08 (529.9)	187.36 (79.06)	1.63 (1.08)	6.38 (1.18) **^, $^	14.54 (10.55)	1.7 (1.39)
CYP3A5 phenotype:							
NM + IM	16	619.19 (347.49)	170.99 (81.41)	1.2 (0.88)	4.5 (1.4)	12.77 (6.8)	2.17 (1.22)
PM	33	808.32 (382.71)	216.46 (92.2)	1.4 (1.02)	5.21 (0.94) *^, $^	11.7 (6.41)	1.59 (0.91)
UGT1A1 phenotype:							
NM	19	837.76 (482.78)	208.06 (103.27)	1.54 (1.04)	4.92 (1.16)	11.26 (6.66)	1.78 (1.31)
IM	22	745.33 (296.5)	203.76 (84)	1.37 (1.03)	5.07 (1.17)	11.36 (5.15)	1.61 (0.73)
PM	8	533.36 (213.91)	180.39 (83.78)	0.76 (0.21) ***	4.88 (1.16)	15.81 (8.69)	2.25 (1.04)

Data are shown as mean (standard deviation). * *p* < 0.05 (*t*-test). ** *p* < 0.05 vs. RM + NM + IM (*t*-test). *** *p* < 0.05 vs. NM (ANOVA). Underlined: *p* < 0.05 in multivariate analysis (with Sex, Race, ABCG2 rs2231142 T/T vs. G/G + G/T, COMT rs13306278 C/T vs. C/C, CYP2B6 PM vs. RM + NM + IM, CYP3A5 PM vs. NM + IM, and UGT1A1 PM vs. NM + IM, *n* = 49). $: *p* < 0.007 after Bonferroni correction in multivariate analysis.

**Table 5:** MS abundances of quetiapine metabolites at t = 2 and t = 10 and observed variations in six healthy volunteers with specific *COMT* rs13306278 genotype and CYP2D6 phenotype.

			Drop in MS Abundance between 2 h and 10 h (%)				
Volunteer	*COMT* rs13306278	CYP2D6	Que. 384.3	NorQue. 296.1	7-OH-Que/Que-SO. 400.2	7.8-diOH-Que *. 416.4	7.8-diOH-N-desal-Que *. 328.3
A	C/C	IM	−8.30	−11.90	−2.80	−2.60	−4.60
B	C/C	NM	−7.50	−1.60	−10.10	7.80	5.00
C	C/T	NM	−21.70	−16.10	−31.80	−11.50	−12.70
D	C/T	IM	−7.60	−12.50	−7.20	−6.10	−3.20
E	C/C	UM	−12.30	−25.70	−22.60	−4.50	−17.20
F	C/T	NM	−10.90	−16.20	−12.10	−0.30	−7.80
Mean			−11.38	−14.00	−14.43	−2.87	−6.75
SD			5.41	7.83	10.77	6.45	7.76
Mean *COMT* rs13306278 C/T			−13.40	−14.93	−17.03	−5.97	−7.90
Mean *COMT* rs13306278 C/C			−9.37	−13.07	−11.83	0.23	−5.60
Mean CYP2D6 UM-NM			−13.10	−14.90	−19.15	−2.13	−8.18
Mean CYP2D6 IM			−7.95	−12.20	−5.00	−4.35	−3.90

Que: quetiapine; OH: hydroxy; SO: sulfoxide; desal: desalkyl. * These metabolites had not been described previously.

## Data Availability

The data presented in this study are available on request from the corresponding author. The data are not publicly available because they belong to the clinical trials’ sponsors.

## Supplementary Materials

The following are available online at https://www.mdpi.com/article/10.3390/pharmaceutics13101573/s1, Table S1. Pharmacokinetic parameters based on CYP2D6 phenotype. Table S2. Pharmacokinetic parameters based on genotypes and phenotypes without statistically significant associations.
