# Supplementary material for: Variants in COMT, CYP3A5, CYP2B6, and ABCG2 Alter Quetiapine Pharmacokinetics
Source: Pharmaceutics. 2021 Sep 28;13(10):1573. doi: 10.3390/pharmaceutics13101573 (PMC8540141; doi:10.3390/pharmaceutics13101573)
Supplement: Supplementary file 1 [file pharmaceutics-13-01573-s001.zip › pharmaceutics-1345190-supplementary.pdf]

pharmaceutics MDPI

Supplementary Materials: Variants in *COMT*, *CYP3A5*, *CYP2B6*, and *ABCG2* Alter Quetiapine Pharmacokinetics

Pablo Zubiaur *, Paula Fernández-Campos, Marcos Navares-Gómez, Paula Soria-Chacartegui, Gonzalo Villapalos-García, Manuel Román, Gina Mejía-Abril, Dolores Ochoa and Francisco Abad-Santos *

Table S1. Pharmacokinetic parameters based on CYP2D6 phenotype.

CYP2D6 Phenotype	n	AUC0-∞/DW (kg ng h/mL mg)	SD	Cmax/DW (kg ng/mL mg)	SD	tmax (h)	SD	t1/2 (h)	SD	Vd/Fw (L/kg)	SD	Cl/Fw (L/h kg)	SD
UM	5	570.24	239.69	224.9	143.35	1.7	1.88	4.85	0.54	14.28	6.82	2.08	1.02
NM	13	716.72	448.37	192.73	86.27	0.99	0.43	4.77	1.11	12.82	8.03	1.93	1.14
IM	28	818.73	366.5	207.04	86.58	1.49	0.96	5.08	1.29	10.59	4.55	1.59	0.97
PM	3	496.15	238.28	150.56	61.11	0.78	0.25	5.13	0.71	18.55	11.87	2.47	1.36
Total	49	746.56	378.69	201.61	90.57	1.34	0.97	4.98	1.14	12.05	6.49	1.78	1.04

UM: ultrarapid metabolizer; NM: normal metabolizer; IM: intermediate metabolizer; PM: poor metabolizer.

Table S2. Pharmacokinetic parameters based on genotypes and phenotypes without statistically significant associations.

			$AUC_{0-\infty}$/DW (kg*ng*h/mL mg)		C_{max}/DW (kg*ng/mL mg)		t_{max} (h)		$t_{1/2}$ (h) (h)		V_d/F_w (L/kg) L/kg		Cl/F_w (L/h*kg)	
Genotype or phenotype		N	Mean	SD	Mean	SD	Mean	SD	Mean	SD	Mean	SD	Mean	SD
ABCB1 rs1128503 *(C1236T)*	CC	19	722.34	360.38	195.60	84.62	1.21	1.08	5.11	0.81	13.11	7.59	1.81	1.01
	CT	20	771.22	426.79	202.28	96.15	1.40	0.83	5.05	1.41	11.18	3.62	1.75	1.05
	TT	10	743.29	343.47	211.68	98.60	1.44	1.09	4.60	1.13	11.77	8.80	1.78	1.20
ABCB1 rs1128503 *(C3435T)*	CC	18	649.45	300.93	182.96	80.42	1.22	1.09	5.00	0.86	13.79	7.48	1.93	0.97
	CT	22	869.78	446.26	218.38	94.91	1.50	0.99	5.06	1.35	10.07	3.68	1.57	1.04
	TT	9	639.59	263.45	197.93	101.30	1.18	0.65	4.74	1.19	13.38	8.86	1.98	1.19
ABCB1 rs2032582 *(G2677T/A)*	GG	14	830.75	395.44	215.02	70.53	1.38	1.28	5.11	0.86	10.76	5.14	1.52	0.80
	GA	19	795.14	437.36	206.88	101.19	1.47	0.92	4.88	1.46	10.69	3.95	1.73	1.08
	GT	8	597.98	260.63	161.49	86.63	1.11	0.83	5.23	0.65	16.01	9.30	2.11	1.15
	AA	7	635.39	290.63	212.06	111.00	1.12	0.70	4.70	1.33	13.78	9.88	2.07	1.34
	AT	1	611.86	0.00	161.42	0.00	1.50	0.00	4.97	0.00	11.89	0.00	1.65	0.00
ABCC2 rs2273697	GG	35	686.31	309.95	197.55	85.00	1.23	0.81	4.86	1.13	12.37	6.53	1.88	1.08
	GA	13	854.30	486.26	204.89	107.06	1.62	1.34	5.04	0.78	11.46	6.77	1.60	0.94
	AA	1	1455.04	0.00	300.92	0.00	1.34	0.00	8.42	0.00	8.44	0.00	0.69	0.00
COMT rs4680	GG	26	711.44	317.25	199.00	98.68	1.43	1.13	4.94	0.83	12.43	6.62	1.79	0.98
	GA	12	798.67	413.23	213.80	76.35	1.20	0.55	5.07	1.54	11.42	7.50	1.69	1.12
	AA	11	772.74	491.61	194.47	91.46	1.28	0.99	4.96	1.39	11.83	5.43	1.86	1.20
*CYP1A2*1B* rs2470890	*1/*1	7	816.67	358.84	197.68	83.48	1.12	0.85	5.32	1.06	11.44	6.10	1.55	0.92
	*1/*1B	22	760.98	463.01	206.62	110.93	1.54	1.25	4.73	1.01	12.83	8.77	1.95	1.31
	*1B/*1B	20	706.17	284.45	197.48	69.79	1.20	0.59	5.14	1.30	11.40	2.87	1.67	0.71
*CYP1A2*1C* rs2069514	*1/*1	27	745.07	437.37	195.08	99.26	1.47	1.11	4.94	1.03	13.23	8.18	1.94	1.24
	*1/*1C	16	755.39	267.94	223.35	79.44	1.13	0.88	4.84	1.11	9.92	2.77	1.50	0.53
	*1C/*1C	6	729.76	407.46	173.02	76.56	1.28	0.23	5.51	1.72	12.40	3.33	1.82	1.08
*CYP1A2*1F* rs762551	*1/*1	25	737.44	347.97	189.63	86.32	1.28	1.05	5.23	1.14	12.69	6.84	1.77	1.00
	*1/*1F	22	753.04	430.54	216.40	98.97	1.37	0.94	4.61	1.10	11.40	6.43	1.83	1.14
	*1F/*1F	2	789.45	279.71	188.62	22.29	1.75	0.35	5.82	0.73	11.23	2.77	1.39	0.53
*CYP2A6*9* rs28399433	*1/*1	39	769.66	406.13	207.07	95.29	1.37	1.03	4.97	1.22	11.96	6.86	1.79	1.11
	*1/*9	6	718.89	253.92	158.63	33.85	1.46	0.83	5.09	0.92	11.07	3.38	1.56	0.57
	*9/*9	4	562.94	210.69	212.87	100.39	0.81	0.14	4.89	0.75	14.35	6.97	2.05	0.98
CYP2C19 phenotype	UM	1	987.23	0.00	204.39	0.00	1.50	0.00	6.34	0.00	9.27	0.00	1.01	0.00
	RM	11	663.42	267.97	200.30	65.50	1.15	0.56	5.06	0.80	13.31	7.16	1.82	0.87
	NM	32	764.12	406.23	199.73	98.99	1.38	1.11	4.83	0.99	11.73	6.77	1.76	1.03
	IM	4	909.53	411.59	258.80	55.46	1.04	0.21	6.37	1.49	11.37	4.12	1.30	0.58
	PM	1	206.75	0.00	44.62	0.00	3.00	0.00	1.98	0.00	13.85	0.00	4.84	0.00

*CYP2C8**2 rs11572103	*1/*1	48	745.05	382.55	197.46	86.69	1.35	0.97	4.98	1.16	12.11	6.54	1.79	1.05
	*1/*2	1	819.30	0.00	400.83	0.00	0.50	0.00	5.07	0.00	8.93	0.00	1.22	0.00
*CYP2C8*3*	*1/*1	37	686.68	334.67	192.16	84.43	1.26	0.88	4.84	1.11	12.20	5.73	1.85	0.97
	*1/*3	11	996.50	417.97	245.88	96.63	1.63	1.28	5.46	1.25	9.55	5.36	1.27	0.80
	*3/*3	1	213.10	0.00	64.21	0.00	1.00	0.00	4.94	0.00	34.01	0.00	4.78	0.00
*CYP2C8*4* rs1058930	*1/*1	48	746.53	382.70	200.82	91.36	1.35	0.98	4.98	1.16	12.10	6.55	1.79	1.05
	*1/*4	1	747.96	0.00	239.36	0.00	0.84	0.00	5.00	0.00	9.64	0.00	1.34	0.00
CYP2C9 phenotype	NM	1	819.30	0.00	400.83	0.00	0.50	0.00	5.07	0.00	8.93	0.00	1.22	0.00
	IM	13	854.22	438.46	217.11	90.81	1.32	0.92	5.51	1.28	12.36	8.05	1.66	1.21
	PM	35	704.50	358.05	190.16	85.30	1.37	1.01	4.78	1.06	12.02	6.01	1.84	1.00
*CYP3A4*22* rs35599367	*1/*1	48	741.55	381.05	201.55	91.53	1.33	0.98	4.95	1.14	12.10	6.54	1.80	1.05
	*1/*22	1	987.23	0.00	204.39	0.00	1.50	0.00	6.34	0.00	9.27	0.00	1.01	0.00
*CYP3A4**3 rs4986910	*1/*1	48	731.80	368.18	199.54	90.35	1.34	0.98	4.91	1.04	12.12	6.53	1.80	1.04
	*1/*3	1	1455.04	0.00	300.92	0.00	1.34	0.00	8.42	0.00	8.44	0.00	0.69	0.00
*SLC22A1**2 rs72552763	*1/*1	27	746.47	397.47	208.56	92.05	1.32	1.06	4.99	1.31	11.96	6.29	1.80	1.10
	*1/*2	21	716.52	343.21	194.07	92.22	1.26	0.75	4.92	0.93	12.42	6.92	1.81	0.99
	*2/*2	1	1380.01	0.00	172.52	0.00	3.50	0.00	6.02	0.00	6.48	0.00	0.74	0.00
*SLC22A1*5* rs34059508	*1/*1	48	749.37	382.18	202.45	91.34	1.33	0.98	4.98	1.16	12.05	6.56	1.78	1.05
	*1/*5	1	611.86	0.00	161.42	0.00	1.50	0.00	4.97	0.00	11.89	0.00	1.65	0.00
SLC28A3 rs7853758	GG	30	788.94	399.27	195.23	92.67	1.46	1.11	5.04	1.22	11.90	6.89	1.79	1.20
	GA	16	685.49	371.27	203.41	82.88	1.19	0.73	4.88	1.12	12.45	6.49	1.80	0.81
	AA	3	648.51	158.90	255.78	125.68	0.86	0.32	4.88	0.49	11.30	2.07	1.63	0.42
SLCO1B1 phenotype	PF	3	775.45	199.81	306.34	141.59	0.61	0.10	4.21	1.01	7.84	1.09	1.37	0.40
	DF	16	793.35	401.20	204.27	85.77	1.13	0.59	5.26	1.05	11.75	6.13	1.64	0.97
	NF	28	733.78	387.39	197.46	81.24	1.46	1.13	5.04	1.08	12.76	7.10	1.81	1.01
UGT1A4 rs2011425	T/T	35	732.18	416.66	193.40	94.93	1.44	1.07	4.98	1.20	12.93	7.39	1.92	1.17
	T/G	13	739.19	226.17	217.67	79.32	1.11	0.65	4.82	0.90	10.01	2.26	1.50	0.47
	G/G	1	1345.98	0.00	280.23	0.00	0.84	0.00	7.00	0.00	7.64	0.00	0.75	0.00
UGT2B15 rs1902023	TT	10	833.41	453.26	219.49	96.80	1.44	1.01	5.11	1.30	10.58	3.52	1.69	1.22
	TG	23	718.71	301.36	196.68	80.20	1.41	1.17	4.89	1.08	12.02	6.94	1.72	0.87
	GG	16	732.32	442.72	197.53	104.53	1.17	0.60	5.02	1.20	13.00	7.35	1.93	1.20

UM: ultrarapid metabolizer; RM: rapid metabolzier; NM: normal metabolizer; IM: intermediate metabolizer; PM: poor metabolizer; NF: normal function; DF: decreased function; PF: poor function.
